# Antimicrobial susceptibility profiles and genotyping of *Neisseria meningitidis* of serogroup C, Italy, 2000–2020

**DOI:** 10.3389/fmicb.2023.1272123

**Published:** 2024-01-03

**Authors:** Paola Vacca, Cecilia Fazio, Arianna Neri, Luigina Ambrosio, Anna Carannante, Florigio Lista, Silvia Fillo, Andrea Ciammaruconi, Antonella Fortunato, Paola Stefanelli

**Affiliations:** ^1^Department of Infectious Diseases, Istituto Superiore di Sanità, Rome, Italy; ^2^Scientific Department, Army Medical Centre of Rome, Rome, Italy

**Keywords:** invasive meningococcal disease, *Neisseria meningitidis*, whole genome sequencing, serogroup C, antimicrobial susceptibility

## Abstract

**Background:**

In Italy the introduction of meningococcal C conjugate vaccine in 2005 has led to a significant reduction of invasive meningococcal disease (IMD) caused by *Neisseria meningitidis* of serogroup C (MenC). However, this serogroup is still responsible of sporadic cases, clusters and local outbreaks. The study aims to investigate the genotype and antimicrobial susceptibility profile of MenC isolates collected in Italy from 2000 to 2020.

**Methods:**

Bacterial isolates and biological samples (blood or cerebrospinal fluid) from invasive meningococcal cases are collected and characterized at the National Reference Laboratory for IMD of Istituto Superiore di Sanità. Antimicrobial susceptibility was determined by MIC Test Strip Method and interpreted according to the EUCAST breakpoints guideline. Genotypic characteristics, including multi locus sequence typing (MLST), finetype, and antimicrobial resistance target genes were performed and analyzed using the PubMLST database. Genomic comparison of core genome MLST (cgMLST) of MenC genomes was also carried out.

**Results:**

From 2000 to 2020, a total of 665 MenC isolates were investigated for antimicrobial susceptibility and 301 for genotyping. Over two decades, almost all MenC isolates resulted susceptible to antimicrobials with few isolates resulting resistant to ciprofloxacin (*N* = 2), penicillin G (*N* = 13), and rifampicin (*N* = 9), respectively. Molecular typing of MenC obtained from isolates or clinical specimens identified mostly the genotype C:P1.5-1,10-8:F3-6:ST-11(cc11). However, phylogenetic analysis, performed on genomes from MenC isolates, identified two sub lineages, 11.1 and 11.2, among cc11, of which the sub lineage 11.2 was the predominant.

**Conclusion:**

Wider application of the genomic analysis and monitoring of antimicrobial susceptibility represent key aspects of IMD surveillance and to monitor the continued evolution of these hyperinvasive strains.

## Introduction

Introduction of meningococcal conjugate vaccine against *Neisseria meningitidis* of serogroup C (MenC) contributed to control the disease after period where many countries had experienced of an increased incidence of invasive meningococcal disease (IMD) due to meningococcal of serogroup C (Pelton, 2016; Acevedo et al., 2019; Read, 2019).

In Italy, vaccination against MenC was firstly introduced into the National Immunization Plan (NIP) 2005–2007,^1^ with a single dose at 13–15 months of age, as recommended vaccination (Stefanelli et al., 2009; Pezzotti et al., 2018).

However, the emergence and the increased spread of hypervirulent MenC strains, particularly those belonging to the clonal complex cc11 (or lineage 11), represent a public health threat, even in those countries where effective treatment and prevention strategies are in place (Lucidarme et al., 2015; Tzeng et al., 2017). Invasive infections caused by meningococci of lineage cc11 (mostly of serogroups C and W, less frequently associated with serogroups B or Y) can be severe, with higher case-fatality rate and high proportion of sequelae (Lucidarme et al., 2015; Caugant and Brynildsrud, 2020). Moreover, events such as capsular switching and adaptation to new niches might contribute to the spread of this genotype (Tzeng et al., 2017; Acevedo et al., 2019; Caugant and Brynildsrud, 2020). Based on genomic analysis, lineage 11 can be split into two sub lineages: 11.1 and 11.2 (Lucidarme et al., 2015). The sub lineage 11.1, includes MenW, MenB and MenC. On the contrary, sub lineage 11.2, harboring the characteristic *fumC* polymorphism, is associated, for the data available so far, exclusively with MenC and MenB (Lucidarme et al., 2015).

The epidemic potential of these hypervirulent strains, which are associated with high rates of mortality and morbidity, together with their ability to modify the antigenic profiles, imposes an ongoing surveillance and genomic analysis (Dickinson et al., 2012; Lucidarme et al., 2015; Caugant and Brynildsrud, 2020).

Nevertheless, serogroup C remains the second capsular serogroup after MenB, as well as the main responsible of local outbreaks occurred in Italy over the years (Fazio et al., 2009; Neri et al., 2015). To date, in Italy, MenC is usually responsible of sporadic cases, small clusters, and regional outbreaks, mostly due to cc11 (Stefanelli et al., 2009, 2012, 2016a, 2016b, 2019).

This study provides a retrospective analysis of MenC causing IMD collected in Italy within an extended surveillance period of 20 years (from 2000 to 2020). Antimicrobial susceptibility phenotypes and their target genes were also investigated. The whole genome sequences were used for genotyping and to identify the genetic lineages. The comparative genomic analysis was performed to identify genetic relatedness of invasive MenC over time.

## Materials and methods

### Collection of isolates and biological samples

All MenC (isolates or biological samples) were collected from 2000 to 2020 within the National Surveillance System of IMD, coordinated by the Istituto Superiore di Sanità (ISS) in collaboration with the Italian Ministry of Health in the framework of DPCM 3/3/2017. The case definition of IMD is based on the EU Commission Decision 2018/945 of 22 June 2018.^2^

Meningococcal isolate serogroups were confirmed by slide agglutination with commercial antisera (Remel Europe, Ltd., UK) or by multiplex PCR (Zhu et al., 2012) on heat-inactivated bacterial suspensions in case of negative or doubtful results by agglutination test. For biological specimens [blood or cerebrospinal fluid (CSF)], meningococcal DNA was extracted using the QiAmp mini kit (Qiagen, Hilden, Germany), following the manufacturer’s procedure. Species identification and genogrouping were performed by RT-PCR using the MenSerogroup kit (Diagenode, Belgium).

### Antimicrobial susceptibility tests

Antimicrobial gradient strip diffusion method was used to define the Minimum Inhibitory Concentrations (MIC) of meningococcal isolates.

Etest (Biomerieux, Sweden) and MIC test strips (Liofilchem, Diagnostici, Italy) were used on Mueller Hinton agar (Oxoid, Ltd., England) plates supplemented with 5% of sheep blood. Meningococcal isolates were tested for a panel of antimicrobials: cefotaxime, ceftriaxone, ciprofloxacin, penicillin G and rifampicin.

MIC values were interpreted according to the clinical breakpoints criteria edited by European Committee Antimicrobial Susceptibility Testing (EUCAST) (v. 13.0).^3^

Primers for the PCR amplification and sequencing used for the main antimicrobial resistance target genes (*gyrA* for ciprofloxacin, *penA* for penicillin G, and *rpoB* for rifampicin) on clinical specimens were performed, as previously described (Taha et al., 2007, 2010; Hong et al., 2013).

### Molecular typing and whole genome sequencing (WGS)

Chromosomal DNA of MenC was extracted using the QiAmp mini kit (Qiagen, Hilden, Germany) from an overnight culture, following the manufacturer’s procedure. WGS was performed only on meningococcal isolates, as previously described (Stefanelli et al., 2016a). PCR and Sanger sequencing were performed on culture-negative meningococcal specimens to determine the genotypes, as previously described (Birtles et al., 2005).

Molecular characterization on MenC was available for those isolates/biological samples collected starting from 2012. Genomes or gene sequences of meningococci were uploaded on PubMLST.org^4^ to obtain the finetype—molecular typing of the variable regions of two outer membrane proteins: VR1 and VR2 of the porin A (PorA) and the variable region of the ferric enterobactin transport (FetA)–and the multi locus sequence typing (MLST).

The combination of capsular group, finetype and MLST defines the genotypic profile, as follows: capsular group: PorA (P1). VR1, VR2: FetA (F)VR: sequence type (ST) (clonal complex).^4^ Alleles of the main antimicrobial resistance target genes–as *gyrA*, encoding the subunit A of DNA gyrase, *penA*, encoding the penicillin binding protein 2 (PBP2), and *rpoB*, encoding for the RNA polymerase β chain–were also analyzed.

Genomic comparison was performed on meningococcal genomes collected between 2012 and 2020, using the Genome Comparator tool available on PubMLST.^5^ The resulting distance matrix was visualized as a Neighbor-Net network in Split Tree4 (version 4.13.1). Incomplete loci were automatically removed from the distance matrix calculation for the neighbor-net graphs. All genome sequences were submitted to PubMLST Neisseria database^4^ (see Supplementary Table 1 for the isolate details). Additionally, a subset of European and non-European genomes belonging to MenC:cc11 (accessible in the *Neisseria* PubMLST^4^) were used for comparison versus Italian MenC:cc11 genomes.

European and non-European genomes were selected according to serogroup, year of isolation, ST, cc and lineages (see Supplementary Table 2 for the isolate details).

## Results

### Antimicrobial susceptibility profile

From 2000 to 2020, 665 MenC isolates were analyzed for antimicrobial susceptibility (Table 1). All meningococci were susceptible to ceftriaxone (MIC ≤ 0.125 mg/L) and to cefotaxime (MIC ≤ 0.125 mg/L). The latter was introduce in the panel of antimicrobials starting from 2014.

**TABLE 1:** Antimicrobial susceptibility of MenC isolates collected in Italy, 2000–2020.

Susceptibility category (MIC) by antimicrobial mg/L)*	Number of isolates per years (N)											
	**2000** **(*N* = 23)**		**2001** **(*N* = 15)**		**2002** **(*N* = 33)**		**2003** **(*N* = 53)**	**2004** **(*N* = 79)**	**2005** **(*N* = 80)**	**2006** **(*N* = 25)**	**2007** **(*N* = 26)**	**2008** **(*N* = 42)**
**Cefotaxime**												
S ≤ 0,125	–		–		–		–	–	–	–	–	–
**Ceftriaxone**												
S ≤ 0,125	23		15		33		53	79	80	25	26	42
**Ciprofloxacin**												
S ≤ 0,03	–		7		–		20	–	24	25	26	42
R > 0,03	–		0		–		0	–	1	0	0	0
**Penicillin G**												
S ≤ 0,25	23		15		33		52	76	77	21	26	42
R > 0,25	0		0		0		1	3	3	4	0	0
**Rifampin**												
S ≤ 0,25	22		15		33		53	78	79	25	26	42
R > 0,25	1		0		0		0	1	1	0	0	0
	**2009** **(*N* = 38)**	**2010** **(*N* = 17)**	**2011** **(*N* = 14)**	**2012** **(*N* = 22)**	**2013** **(*N* = 26)**	**2014** **(*N* = 25)**	**2015** **(*N* = 32)**	**2016** **(*N* = 40)**	**2017** **(*N* = 29)**	**2018** **(*N* = 19)**	**2019** **(*N* = 21)**	**2020** **(*N* = 6)**
**Cefotaxime**												
S ≤ 0,125	–	–	–	–	–	25	32	40	29	19	21	6
**Ceftriaxone**												
S ≤ 0,125	38	17	14	22	26	25	32	40	29	19	21	6
**Ciprofloxacin**												
S ≤ 0,03	38	16	14	22	26	25	32	40	29	19	21	6
R > 0,03	0	1	0	0	0	0	0	0	0	0	0	0
**Penicillin G**												
S ≤ 0,25	37	17	14	22	26	25	32	40	29	19	20	6
R > 0,25	1	0	0	0	0	0	0	0	0	0	1	0
**Rifampin**												
S ≤ 0,25	35	17	14	21	26	25	32	40	28	19	20	6
R > 0,25	3	0	0	1	0	0	0	0	1	0	1	0

*According to the new EUCAST breakpoints (v 13.0).

According to the EUCAST breakpoints,^3^ 98% (648/665) of isolates were susceptible to penicillin G (MIC_*S*_ < 0.25 mg/L); however, more than half showed MIC values close to the resistance breakpoint (MIC values between 0.094 and 0.25 mg/L).

Thirteen MenC were penicillin G resistant (MIC > 0.25 mg/L) with MIC values ranging from 0.38 to 0.50 mg/L. Analysis of *penA* gene revealed 16 alleles, of which 10 were characterized by polymorphisms in the C-terminal region of PBP2 (F504L, A510V, I515V, H541N, and I566V) and associated with reduced susceptibility to penicillin G. The remaining 6 alleles were considered wild-type because no alteration was present in PBP2 and were associated with susceptibility to penicillin G. Among the *penA* alleles found in this study, 4 isolates harbored *penA327* having only 4 out of 5 alterations (F504L, A510V, I515V, H541N) in the corresponding amino acid sequence. These isolates showed MIC values ranging between 0.094 and 0.25 mg/L.

Overall, the *penA248* (*N* = 100) was the most frequent allele associated with cc11 sub lineages.

Ciprofloxacin was introduced in the antimicrobial susceptibility test starting from 2005. Except for 2 isolates, resistant to ciprofloxacin (MIC values of 0.064 and 0.094 mg/L, respectively), the remaining were susceptible.

Molecular investigation of the target gene *gyrA* identified *gyrA4* (*N* = 182), *gyrA18* (*N* = 31), *gyrA2* (*N* = 4), *gyrA5* and *gyrA12* (one isolates each one), as the main alleles.

Although the majority of isolates were susceptible to rifampicin, 9 MenC isolates resulted resistants (MIC > 0.25 mg/L), showing MIC values of 0.38 mg/L (*N* = 3), 0.5 mg/L (*N* = 1), 32 mg/L (*N* = 3), and 256 mg/L (*N* = 2), respectively. The presence of the amino acid substitution H552Y, in the *rpoB* gene, confirmed the antimicrobial resistant phenotype to rifampicin.

### Genotyping

Genotyping was performed on 301 MenC of which 232 isolates and 69 biological samples, collected from 2012 to 2020 (Table 2). Overall, MenC were grouped in 13 ccs. Cc11 (248/301; 82%) was the predominant over the years, of them 94% (234/248) belonged to ST-11. Cc334 was the second most frequent clonal complex (34/301; 11%), mostly of ST-1031 (27/34). The remaining ccs were cc8, cc18, cc22, cc32, cc103, cc175, cc198, cc231, cc269, cc865, and cc10217. Four MenC belonged to ccs not assigned at the time of writing (unknown, UNK).

**TABLE 2:** Genotypic profile of invasive MenC, both from microbiological isolates and clinical specimens, collected in Italy from 2012 to 2020.

Clonal complex (cc) (N°)	Sequence Type (ST)	PorA VR1	PorA VR2	FetA VR	N°
cc11 (*N* = 248)	ST-11	5	2	F1-5	1
		5	2	F3-3	23
		5-1	10-1	F3-6	2
		5-1	10-6	F3-3	1
		5-1	10-8	F3-6	197
		5-1	10-8	F1-12	1
		5-1	10-8	NA	2
		ND	ND	F3-6	4
		ND	10-8	F1-5	1
		ND	ND	ND	1
		ND	ND	F3-3	1
	ST-1190	5	2	F3-6	1
	ST-2780	5-1	10-8	F3-6	2
	ST-3463	5-1	10-8	F3-6	1
	ST-11760	5-1	10-8	F3-6	3
	ST-12051	5-1	10-8	F3-6	1
	ST-11936	5-1	10-8	F3-6	1
	ST-13254	5-1	10-8	F3-6	1
	ND	ND	ND	ND	4
**cc334 (*N* = 34)**	ST-1031	22	14-6	F3-9	1
		22	14	F3-9	1
		5-1	10-8	F3-9	1
		7-4	14-6	F3-6	1
		7-4	14-6	F3-9	23
	ST-10134	7-4	14-6	F3-9	4
	ST-11168	7-4	14-6	F3-9	1
	ST-12744	7-4	14-6	F3-9	1
	ST-15568	7-4	14-6	F3-9	1
**UNK (*N* = 4)**	ST-8416	5-2	10-1	F1-7	1
	ST-10134	7-4	14-6	F3-9	1
	ST-11584	22	26	F1-14	1
	ST-11337	17	16-26	F3-3	1
**cc32 (*N* = 4)**	ST-561	19-11	15	F5-1	1
	ST-32	7	16-26	F3-3	1
	ST-7460	7-1	1	F3-3	2
**cc8**	ND	5	2	ND	1
**cc18**	ST-5029	5-2	10	F5-42	1
**cc22**	ST-184	5-1	10-2	F4-1	1
**cc103**	ST-5133	18-1	3	F3-9	1
**cc175**	ST-5770	5-1	10-1	F1-7	1
**cc198**	ST-823	7-2	13-5	F1-150	1
**cc231**	ST-11583	5-1	2-2	F5-7	1
**cc269**	ST-467	5-1	15-11	F1-7	1
**cc865**	ST-3327	21	16-36	F5-5	2
**cc10217**	ND	21-15	16-50	F1-7	1

ND: Not determined.

The finetype combination (PorA VR1, VR2: FetA VR) P1.5-1,10-8:F3-6 (*N* = 206), followed by P1.7-4,14-6: F3-9 (*N* = 30), P1.5,2: F3-3 (*N* = 23) was the most common.

Overall, 37 genotypes were found and C:P1.5-1,10-8:F3-6:ST-11(cc11) (*N* = 197), C:P1.5,2:F3-3:ST-11(cc11) (*N* = 23), and C:P1.7-4,14-6:F3-9:ST-1031(cc334) (*N* = 23) resulted the prevalent.

### Comparative genomic analysis

**FIGURE 1:**
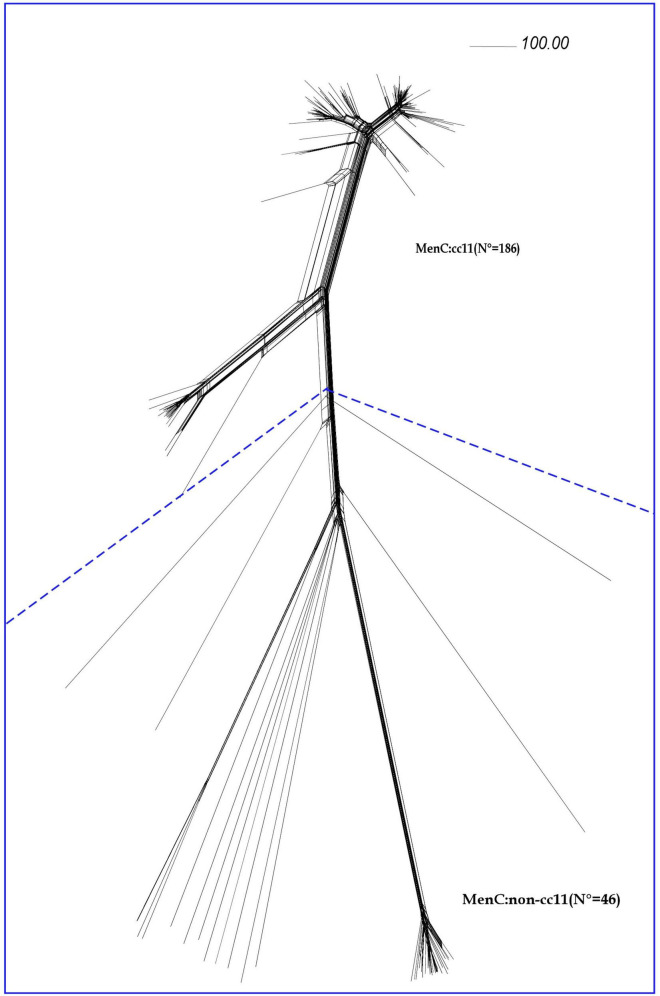
Core genome analysis by cgMLST on MenC genomes. Neighbor-net phylogenetic network based on a comparison of 1605 core genome loci of genomes from invasive meningococcal isolates of serogroup C (MenC) belonging to cc11 (*N* = 186) and to different ccs (MenC:non-cc11), (*N* = 46). The scale bar represents a pairwise allelic difference of 100.

Comparative genomic analysis was performed on 232 meningococcal isolates collected between 2012 and 2020 (Figure 1).

According to cgMLST analysis, all the MenC genomes belonging to cc11 (*N* = 186) clustered in lineage 11, separated from the MenC genomes belonging to others ccs (MenC:non-cc11) (*N* = 46).

**FIGURE 2:**
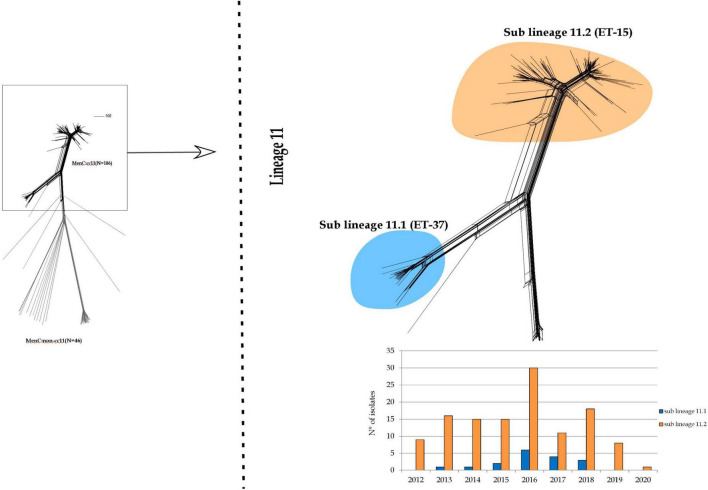
Core genome analysis by cgMLST on MenC:cc11. Invasive MenC belonged to cc11 clustering in a single main lineage (lineage 11) spitting into two sub lineages: 11.1 and 11.2.

As observed in Figure 2, lineage 11 split into two sub lineages: 11.1 and 11.2. The latter was prevalent during the entire study period.

**FIGURE 3:**
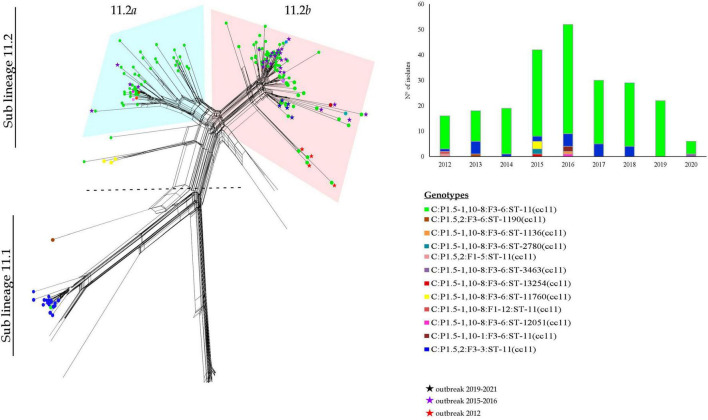
Core genome analysis by cgMLST on MenC:cc11 sub lineages 11.1 and 11.2 and temporal trend.

Sub lineage 11.1 grouped strains from sporadic IMD cases occurred between 2013 and 2018. Except for one, all MenC genomes belonging to sub lineage 11.1 were genotyped as C:P1.5,2:F3-3:ST-11(cc11) (Figure 3).

All the genomes belonging to sub lineage 11.2 carried the *fumC* 640 G>A polymorphism characteristic of the ET-15 meningococci, and genotyped as C:P1.5-1,10-8:F3-6:ST-11(cc11) (Figure 3).

The sub lineage 11.2 can be distinct in two groups, here called 11.2*a* and 11.2*b*.

The 11.2*a* group included 51 genomes from isolates collected from 2012 to 2018. In particular, the majority of them belonged to the genotype C:P1.5-1,10-8:F3-6:ST-11(cc11), except for 2 MenC:cc11 with different finetypes—P1.5-1,10-8:F1-12 and P1.5-1,10-1:F3-6. Moreover, in this group clustered also 7 isolates non-ST-11, specifically ST-12051 (*N* = 1), ST-2780 (*N* = 2), ST-13254 (*N* = 1), ST-11760 (*N* = 3) (Figure 3).

Among the genomes in the 11.2*a* group, 47 genomes were identified in strains responsible of sporadic cases and 4 genomes from an outbreak occurred between 2015 and 2016.

The 11.2*b* group comprised 100 genomes from isolates collected from 2012 to 2020, and accounted the majority of meningococci circulating in Italy during the last years. Sixty of them were from sporadic IMD cases between 2012 and 2019, whereas 40 were from outbreaks occurred in Italy between the years 2012 and 2020 (Stefanelli et al., 2012, 2016b, 2019; Fazio et al., 2022). Genotype C:P1.5-1,10-8:F3-6:ST-11(cc11) was the predominant, 3 genomes showed different STs, ST-13254 (*N* = 1) and ST-2780 (*N* = 2). As shown in Figure 3, the majority of MenC of recent isolation, belonging to the sub lineage 11.2, was at the more distal region of the lineage.

**FIGURE 4:**
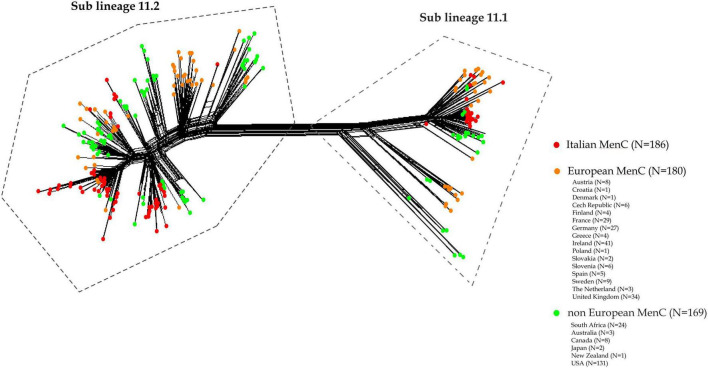
Core genome analysis by cgMLST on a subset of MenC:cc11 lineage 11 meningococcal genomes from Italy, European and non-European countries. Genetic relatedness of the Italian MenC:cc11 lineage 11 (*N* = 186) versus a subset of genomes collected worldwide during the same time period (*N* = 169, non-European; *N* = 180 European).

As shown in Figure 4, (for isolates information see Supplementary Tables 1, 2), genetic relatedness was carried out between MenC:cc11 lineage 11 genomes from this study and a subset of genomes of MenC lineage 11 collected worldwide in the same time period available in the PubMLST database. Based on genomic comparison, all the genomes clustered according to the cc11 lineage structure as described by Lucidarme et al. (2015). In both sub lineages 11.1 and 11.2, Italian MenC were mostly intermixed with European isolates (Figure 4). Among the non-European isolates, the genome sequences from USA were more similar to the Italian MenC (Figure 4).

## Discussion

Invasive meningococcal disease caused by MenC remains a public health concern due to the rapid onset and progression of the disease with fatal outcome and possible sequelae (Caugant and Brynildsrud, 2020). Italy ranks among low IMD incidence European countries of IMD (specifically 0.12 cases per 100 000 inhabitants in 2020) with a predominance of MenB, followed by MenC.^6^

The introduction of antimeningococcal C conjugated vaccine into the Italian NIP 2005–2007 contributed in the reduction of IMD due to this serogroup (Neri et al., 2015; Pezzotti et al., 2018) in the following years, mostly among children for whom the vaccination is targeted and recommended (Neri et al., 2015).

Although in Italy most of the MenC IMD cases occur sporadically, clusters and local outbreaks caused by hypervirulent C:P1.5-1,10-8,F3-6:ST-11(cc11) have been reported (Fazio et al., 2009; Stefanelli et al., 2012, 2016b). A large cluster of MenC:cc11 in Tuscany contributed to the increase of cases in the country (Stefanelli et al., 2016a,b) up to 44 and 43% of all the IMD cases occurred in 2015 and 2016, respectively (Pezzotti et al., 2018). In 2018, a local outbreak was identified in Sardinia due to a switched strain MenB:cc11 (Stefanelli et al., 2019). Between December 2019 and January 2020, an outbreak caused by MenC:cc11 occurred in a limited area in the Northern part of Italy (Fazio et al., 2022).

Outbreaks caused by MenC:cc11 have also been reported in the past in some European countries (De Schrijver and Maes, 2003; Ali et al., 2014; Taha et al., 2016) and the USA (Tzeng et al., 2017). In 2015, it was reported the first large-scale outbreak of MenC in Africa (Nnadi et al., 2017; Bozio et al., 2018), caused by the spread of a new MenC strain belonging to cc10217 (De Schrijver and Maes, 2003; Nnadi et al., 2017).

Although antimicrobial resistance in *N. meningitidis* does not currently represent an issue, resistant isolates were reported worldwide, mostly against antimicrobials used for prophylaxis (Acevedo et al., 2019). In agreement with that reported in others countries (Acevedo et al., 2019), in Italy meningococcal isolates resistant to penicillin, ciprofloxacin or rifampicin are rare, here representing the 2, 0.3 and 0.9%, respectively.

Molecular profiling of *gyrA* target gene for ciprofloxacin resistance showed mutations particularly at T91I amino acid position. Ciprofloxacin-resistance strains have also been described in other European countries, the USA, South America and in China (Acevedo et al., 2019). Resistance to rifampicin is correlated to the alterations in the *rpoB* gene. However, strains that harbor a modified *rpoB* gene are rare, identified in Europe and also in our country (Acevedo et al., 2019). Rifampicin resistant meningococci represent a great concern, especially in those countries where rifampicin is used as the first-line choice for chemoprophylaxis (Acevedo et al., 2019). *PenA* gene mutations and mosaicism represent the main molecular mechanisms associated with penicillin resistance (Taha et al., 2007).

Overall, in this study, 82% of MenC belonged to the genotype C:P1.5-1,10-8:F3-6:ST-11(cc11).

The core genome comparison of MenC, here analyzed, confirmed the phylogenetic structure of cc11 lineage 11, as described by Lucidarme et al. (2015), with two main sub-lineages: 11.1 and 11.2. Here, the majority of MenC belonged to the sub lineage 11.2, comprising genomes of strains causing both sporadic IMD cases or outbreaks.

Based on our previously study on MenC strains circulating in Italy (Lo Presti et al., 2019), using a Bayesian method to reconstruct the epidemiological dynamics, the C:P1.5-1,10-8:F3-6:ST-11(cc11) genotype segregated into two distinct clades, both apparently originating from UK, but in a different time periods. The study suggested a possible first introduction in our country between 2007 and 2011, where this variant was spreading among several European countries (Lo Presti et al., 2019). The second possible introduction could be reconducted between 2013 and 2014 (Lo Presti et al., 2019). It is likely that this strain has spread in Italy and remains stable as suggested by genomic and phylogenetic analysis, here reported.

In conclusion, this study provides a comprehensive characterization on antimicrobial susceptibility profiles and genetic characteristics of invasive meningococcal serogroup C collected over 20 years. Comparative genomic analysis on MenC isolates has provided valuable information on MenC population structure identifying the hypervirulent lineages and also on clusters and outbreaks occurred in our country. These results highlight the need to continue an active IMD surveillance to monitor the emergence and persistence of particular meningococci, such as those belonging to the cc11 lineage and related sub lineages and to compare them with those identified worldwide.

## Data availability statement

The data presented in this study are deposited in online repositories (https://pubmlst.org/bigsdb?db=pubmlst_neisseria_isolates&page=plugin&name=Export). Accession numbers can be found in the Supplementary material.

## Author contributions

PV: Conceptualization, Data curation, Formal analysis, Investigation, Methodology, Software, Writing – original draft. CF: Formal analysis, Writing – review and editing. AN: Formal analysis, Writing – review and editing. LA: Writing – review and editing. ACa: Writing – review and editing. FL: Writing – review and editing. SF: Writing – review and editing. ACi: Writing – review and editing. AF: Writing – review and editing. PS: Conceptualization, Supervision, Validation, Visualization, Writing – review and editing.

## Footnotes

1
https://www.salute.gov.it/portale/documentazione/p6_2_2_1.jsp?id=543&lingua=italiano

2
https://eur-lex.europa.eu/legal-content/EN/TXT/PDF/?uri=CELEX:32018D0945

3
http://eucast.org/clinicalbreakpoints/

4
http://pubmlst.org/neisseria/

5
https://pubmlst.org/bigsdb?db=pubmlst_neisseria_isolates&page=plugin&name= Genome Comparator

6
https://www.iss.it/sn-mbi-rapporti-iss
